# CA-MRSA and HA-MRSA coexist in community and hospital settings in Uganda

**DOI:** 10.1186/s13756-019-0551-1

**Published:** 2019-06-03

**Authors:** David Patrick Kateete, Freddie Bwanga, Jeremiah Seni, Raymond Mayanja, Edgar Kigozi, Brian Mujuni, Fred K. Ashaba, Hannington Baluku, Christine F. Najjuka, Karin Källander, Elizeus Rutebemberwa, Benon B. Asiimwe, Moses L. Joloba

**Affiliations:** 1 Department of Immunology and Molecular Biology, Makerere University College of Health Sciences, Kampala, Uganda; 2 Department of Medical Microbiology, Makerere University College of Health Sciences, Kampala, Uganda; 3 Catholic University of Health and Allied Sciences – Bugando, Mwanza, Tanzania; 4 Makerere University Walter Reed Project, Kampala, Uganda; 5 Malaria Consortium, London, UK; 6 Department of Public Health Sciences, Karolinska Institutet, Stockholm, Sweden; 7 Makerere University School of Public Health, Kampala, Uganda

**Keywords:** Eastern Uganda, Iganga/Mayuge districts, Coexistence, Hospital-associated MRSA, Community-associated MRSA, *mecA*, SCC*mec* types, *spa* types

## Abstract

**Background:**

Methicillin resistant *Staphylococcus aureus* (MRSA) strains were once confined to hospitals however, in the last 20 years MRSA infections have emerged in the community in people with no prior exposure to hospitals. Strains causing such infections were novel and referred to as community-associated MRSA (CA-MRSA). The aim of this study was to determine the MRSA carriage rate in children in eastern Uganda, and to investigate coexistence between CA-MRSA and hospital-associated (HA-MRSA).

**Methods:**

Between February and October 2011, nasopharyngeal samples (one per child) from 742 healthy children under 5 years in rural eastern Uganda were processed for isolation of MRSA, which was identified based on inhibition zone diameter of ≤19 mm on 30 μg cefoxitin disk. SCC*mec* and *spa* typing were performed for MRSA isolates.

**Results:**

A total of 140 *S. aureus* isolates (18.9%, 140/742) were recovered from the children of which 5.7% (42/742) were MRSA. Almost all (95.2%, 40/42) MRSA isolates were multidrug resistant (MDR). The most prevalent SCC*mec* elements were types IV (40.5%, 17/42) and I (38.1%, 16/42). The overall frequency of SCC*mec* types IV and V combined, hence CA-MRSA, was 50% (21/42). Likewise, the overall frequency of SCC*mec* types I, II and III combined, hence HA-MRSA, was 50% (21/42). Spa types t002, t037, t064, t4353 and t12939 were detected and the most frequent were t064 (19%, 8/42) and t037 (12%, 5/42).

**Conclusion:**

The MRSA carriage rate in children in eastern Uganda is high (5.7%) and comparable to estimates for Mulago Hospital in Kampala city. Importantly, HA-MRSA (mainly of *spa* type t037) and CA-MRSA (mainly of *spa* type t064) coexist in children in the community in eastern Uganda, and due to high proportion of MDR detected, outpatient treatment of MRSA infection in eastern Uganda might be difficult.

**Electronic supplementary material:**

The online version of this article (10.1186/s13756-019-0551-1) contains supplementary material, which is available to authorized users.

## Background

*Staphylococcus aureus* is a recognized cause of mild to severe infections worldwide [1, 2]. However, the burden due to staphylococcal infections in Africa is apparently overshadowed by the ‘big three’ diseases –HIV/AIDS, tuberculosis and malaria [3, 4]. Indeed, surveillance studies on bacterial infections in Africa show that *S. aureus* is a common pathogen in healthy adults and immunosuppressed individuals [3–12], and persons with genetic predispositions [10]. As well, *S. aureus* is second only to the pneumococcus among the frequent causes of pneumonia in children in Africa [13].

Methicillin resistant *S. aureus* (MRSA) strains, now widespread globally, have complicated treatment and control of staphylococcal infections. Once confined to hospitals and/or health care environments, MRSA strains are now frequent causes of infection in the community. Nevertheless, surveillance studies have revealed differences in MRSA strains causing infections in hospitalized patients and healthcare workers in hospitals vis-à-vis MRSA isolates causing infection in the community (i.e. community-associated MRSA) [1, 2, 14]. While such demarcation of MRSA as “hospital/health care-associated MRSA” (HA-MRSA) or “community-associated MRSA” (CA-MRSA) can be confusing [1, 14–16], there are clear differences in phenotypes and genetic background of MRSA strains associated with infection in either setting, community or hospital [1, 2, 14, 17, 18]. Genotypically, CA-MRSA are newer and more virulent strains, which emerged in the late 1990s as major causes of skin and soft tissue infections in healthy and relatively young people with no prior exposure to hospitals [1, 2, 19, 20]. CA-MRSA strains typically carry SCC*mec* types IV or V and they are generally susceptible to non-β-lactam antimicrobials [1, 2, 14]. Additionally, CA-MRSA carry (but not always) Panton Valentine Leukocidin (PVL) encoding genes *LukS-PV* and *LukF-PV* [1, 2] that are associated with increased virulence. On the other hand, HA-MRSA strains carry SCC*mec* types I, II, or III and seldom possess PVL-encoding genes [1, 2]. HA-MRSA are associated with nosocomial infections e.g. endocarditis and they are often resistant to non-β-lactam antimicrobial agents especially aminoglycosides, macrolides, lincosamides and fluoroquinolones [1, 2].

Although CA-MRSA has been predicted to replace HA-MRSA in hospitals [19], mathematical models predict coexistence between the two strains given the high rates of discharge and hospitalization which bolster hospital-community interactions [21]. Relatedly, we previously detected MRSA strains carrying SCC*mec* types IV or V at Mulago Hospital in Kampala city [22, 23], pointing to coexistence between CA-MRSA and HA-MRSA in the hospital. The aim of this study was to determine the MRSA carriage rate in children in eastern Uganda, and to investigate coexistence between CA-MRSA and HA-MRSA.

## Methods

### Study setting, susceptibility testing and MRSA identification

This cross-sectional study was nested in a study that investigated pneumococcal carriage in children under 5 years age in the Iganga/Mayuge Health & Demographic Surveillance Site (IMHDSS) [13], located in Iganga and Mayuge districts in eastern Uganda 120 km from Kampala city, Uganda’s capital. The IMHDSS is a rural community covering a contiguous area of ~ 155 km^2^ comprising of 65 villages and approx. 85,000 people living in 15,652 households. It is characterized with significant interaction between healthcare workers and community members, and 13% of its population are children under 5 years of age [24].

The children screened for MRSA carriage were from a total of 742 households (one child per household). They were selected from the IMHDSS population register using simple random sampling. Each household was visited and a child aged between 2 months and 59 months was selected using the lottery method of sampling. Using a pretested questionnaire, the primary caretaker of the child in each household was interviewed for information on demographic characteristics, history of illnesses and antibiotic treatment. Following caretaker consent, a nasopharyngeal sample from each child was collected by a study nurse using pre-packed sterile calcium alginate swabs on flexible aluminum shafts (Becton, Dickson and Company, New Jersey). Swabs were placed in Amies transport medium in a tube and transported to the Clinical Microbiology laboratory at Makerere University College of Health Sciences where they were processed for culturing and isolation/identification of *S. aureus* according to standard microbiological procedures published previously [25].

Susceptibility of *S. aureus* to antibiotics was determined by the disc diffusion antibiotic sensitivity testing method as recommended by the Clinical and Laboratory Standards Institute, (CLSI, 2011) [26]. Briefly, colonies of pure bacterial isolates were suspended in sterile normal saline to a turbidity of McFarland standard 0.5, and uniformly spread on Muller Hinton agar (MHA) plates (Biolabs®, Hungary) with antibiotic disks (Biolabs®, Hungary): penicillin G (10 U), cefoxitin (30 μg), clindamycin (2 μg), erythromycin (15 μg), vancomycin (30 μg), tetracycline (30 μg), linezolid (30 μg), trimethoprim/sulphamethoxazole (1.25/23.5 μg), chloramphenicol (5 μg), ciprofloxacin (5 μg), and gentamicin (10 μg). Plates were incubated at 37 °C for 24 h. Inhibition zones were measured in millimeters and interpreted as susceptible (S), intermediate (I) or resistant (R). *S. aureus* with inhibition zone diameters of ≤19 mm on 30 μg cefoxitin disk were considered to be MRSA, and confirmed for *mecA* gene carriage by PCR [1, 22].

### Classification of isolates as CA-MRSA or HA-MRSA

Because CA-MRSA and HA-MRSA can be recovered from either setting (i.e. community or hospital), to classify isolates as CA-MRSA or HA-MRSA we used isolate genotypic characteristics and not clinical/epidemiological features. Thus, given that SCC*mec* types I, II & III are typically restricted to HA-MRSA and not found widely in healthy populations while SCC*mec* types IV & V are predominantly associated with CA-MRSA [1, 2, 18, 19, 21, 27], MRSA isolates that carried SCC*mec* types I, II, or III were classified as HA-MRSA while isolates with SCC*mec* types IV or V were classified as CA-MRSA [28]. SCC*mec* genotyping to delineate HA-MRSA and CA-MRSA was performed as described by Boye, et al., (2007) [29]. Also, we compared SCC*mec* types for MRSA isolates from the IMHDSS in eastern Uganda to previously described SCC*mec* types for MRSA isolates from Mulago Hospital in Kampala [22, 23] and pastoral communities in rural western Uganda [30, 31]. Coexistence between HA-MRSA and CA-MRSA in the community or hospital was based on occurrence of MRSA strains with genetic background of both HA-MRSA (i.e. SCC*mec* types I, II or III) and CA-MRSA (i.e. SCC*mec* types IV or V) in either setting.

### Spa typing

For spa typing, the *x*-region (200–400 bp) of *S. aureus spa* gene was amplified from MRSA with primers and PCR conditions described by Harmsen et al., 2003 [32]. Purified PCR products were sequenced at MBN Laboratories (Kampala, Uganda) or ACGT Inc. (Wheeling, IL, USA) using forward and reverse primers used in PCRs. To obtain *spa* types, sequences were submitted to an online spaTyper server (http://spatyper.fortinbras.us/) and confirmed by cross-checking with Ridom Spa Server (http://spaserver2.ridom.de/spatypes.shtml). For quality control, standard reference *S. aureus* strains ATCC-43300 -*mecA*+, PVL- (MRSA) and ATCC-29213 -mecA-, PVL- (MSSA) & ATCC-25923 -*mecA*-, PVL+ (MSSA) were used as positive or negative controls. Furthermore, to detect *PVL* genes, isolates were subjected to PCR-detection of a 433 bp fragment overlapping the *lukS-PV* and *lukF-PV* genes using previously published protocols [22, 23]. Apart from *spa* typing in which all PCR products were sequenced, DNA sequencing of amplified segments of *mecA* and *PVL* genes for randomly selected isolates was performed and sequences confirmed by BLAST searching at the National Center for Biotechnology Information (NCBI) https://blast.ncbi.nlm.nih.gov/Blast.cgi

## Results

### Demographics

MRSA isolates were recovered from 42 of the 742 children sampled. The characteristics of study population were described previously [13] however, we will highlight a few statistics pertinent to this study. The mean age of the children was 30 months and 52% were girls. All children were healthy at the time of screening i.e. none had observable clinical symptoms however, based on reports of caretakers (mothers), majority (≥90%) were sick 2 weeks prior to screening and the most common symptoms were fever, running nose and cough. Approx. 30% of the previously sick children were given antibiotics, mostly ampicillin and co-trimoxazole.

### MRSA prevalence and drug resistance patterns

The processed nasopharyngeal samples yielded 600 Gram positive and catalase positive isolates (one per sample/child) of which 140 were confirmed to be *S. aureus*. Thirty per cent (42/140) of *S. aureus* were cefoxitin resistant and these were confirmed to be MRSA upon *mecA* gene PCR (all 42 isolates were *mecA* positive). Thus, MRSA prevalence in *S. aureus* isolates was 30% and its carriage rate in children was 5.7% (42/742). Almost all MRSA isolates i.e. 95.2% (40/42) were multidrug resistant (MDR, resistance to three or more classes of antimicrobials) and MDR rates for CA-MRSA and HA-MRSA isolates were similar, Table 1. All MRSA isolates were susceptible to rifampicin and anti-MRSA agents (vancomycin & linezolid) and generally to clindamycin but they were significantly resistant to non-β-lactam antimicrobial agents commonly used to treat staphylococcal infections (SXT, erythromycin, gentamicin, chloramphenicol).

**Table 1:** Genotypes and antibiotic susceptibility patterns of CA- & HA-MRSA from children ≤5 years in eastern Uganda

Isolate #	PVL	*mecA*	SCC*mec*	FOX	PEN	TET	ERY	SXT	CHL	GEN	CIP	CLI	RIF	VAN	LZD	MDR phenotype	*Spa* type
HA-MRSA (*n* = 21)																	
52–1	+	+	I	R	R	R	R	R	R	R	S	S	S	S	S	Yes	
1320–1	–	+	I	R	R	R	R	R	R	R	R	S	S	S	S	Yes	
244C-1	–	+	I	R	R	R	R	R	R	R	S	S	S	S	S	Yes	
K1057–1	–	+	I	R	R	R	S	S	R	R	S	S	S	S	S	Yes	**t037**
K264–1	+	+	I	R	R	R	S	S	R	R	S	S	S	S	S	Yes	
K284–1	–	+	I	R	R	R	R	R	R	R	S	S	S	S	S	Yes	
K36–1	–	+	I	R	R	R	R	R	R	S	S	S	S	S	S	Yes	**t037**
K370–1	–	+	I	R	R	R	R	R	R	R	S	S	S	S	S	Yes	
K39–1	–	+	I	R	R	R	R	R	R	R	R	S	S	S	S	Yes	
K4834–1	–	+	I	R	R	R	S	S	R	R	S	S	S	S	S	Yes	**t037**
K970–1	–	+	I	R	R	R	S	I	S	S	I	S	S	S	S	No	**t037**
R030–1	–	+	I	R	R	R	R	R	R	R	R	S	S	S	S	Yes	
R10–1	–	+	I	R	R	R	R	R	R	S	R	S	S	S	S	Yes	t12939
R110–1	+	+	I	R	R	R	R	R	S	S	S	S	S	S	S	Yes	Unknown
R19–1	–	+	I	R	R	R	R	R	R	R	S	S	S	S	S	Yes	
R220–1	–	+	I	R	R	R	R	R	S	R	S	S	S	S	S	Yes	Unknown
1322–1	+	+	II	R	R	R	R	R	R	R	I	I	S	S	S	Yes	
306C-1	–	+	II	R	R	R	R	R	I	R	S	S	S	S	S	Yes	
K911–1	–	+	II	R	R	R	R	S	R	R	R	S	S	S	S	Yes	
R160–1	–	+	II	R	R	R	R	R	S	S	S	S	S	S	S	Yes	t002
R17–1	–	+	III	R	R	R	S	R	S	I	S	S	S	S	S	Yes	**t037**
Total +/R HA-MRSA (%)	4 (19)	21 (100)	21 (100)	21 (100)	21 (100)	21 (100)	16 (76.2)	16 (76.2)	15 (71.4)	15 (71.4)	5 (23.8)	0 (0)	0 (0)	0 (0)	0 (0)	20 (95.2)	
CA-MRSA (*n* = 21)																	
1325–1	–	+	IV	R	R	R	R	R	R	I	S	S	S	S	S	Yes	Unknown
1326–1	+	+	IV	R	R	R	R	S	S	S	R	S	S	S	S	Yes	
K2240–1	–	+	IV	R	R	S	S	S	S	S	R	I	S	S	S	No	t4353
K2810–1	–	+	IV	R	R	R	R	R	S	S	R	S	S	S	S	Yes	
R31B-1	–	+	IV	R	R	R	R	R	S	R	R	S	S	S	S	Yes	**t064**
R310–1	–	+	IV	R	R	R	R	R	R	R	S	S	S	S	S	Yes	**t064**
R33–1	+	+	IV	R	R	R	R	R	R	R	S	S	S	S	S	Yes	**t064**
K3700–1	–	+	IV	R	R	R	S	S	R	R	S	S	S	S	S	Yes	
R0100–1	–	+	IV	R	R	R	R	R	S	S	S	S	S	S	S	Yes	**t064**
R02–1	–	+	IV	R	R	R	R	R	R	S	R	S	S	S	S	Yes	Unknown
R020–1	–	+	IV	R	R	S	S	R	R	R	S	S	S	S	S	Yes	
R0300–1	–	+	IV	R	R	R	R	R	R	S	S	S	S	S	S	Yes	
R06–1	–	+	IV	R	R	R	R	R	S	R	S	S	S	S	S	Yes	**t064**
R08–1	+	+	IV	R	R	R	R	R	R	R	S	S	S	S	S	Yes	**t064**
R18–1	–	+	IV	R	R	R	R	S	R	S	R	S	S	S	S	Yes	
R20–1	–	+	IV	R	R	I	R	R	R	R	S	S	S	S	S	Yes	**t064**
R26A-1	–	+	IV	R	R	R	R	R	S	S	R	S	S	S	S	Yes	**t064**
R040–1	–	+	V	R	R	R	R	R	R	R	R	S	S	S	S	Yes	
K60–1	+	+	V	R	R	R	R	R	S	S	R	S	S	S	S	Yes	
K38–1	–	+	V	R	R	R	R	R	R	R	I	S	S	S	S	Yes	
K350C-1	+	+	V	R	R	R	S	S	S	S	R	S	S	S	S	Yes	Unknown
Total +/R CA-MRSA (%)	5 (24)	21 (100)	21 (100)	21 (100)	21 (100)	18 (85.7)	17 (81)	16 (76.2)	12 (57.1)	10 (47.6)	10 (47.6)	0 (0)	0 (0)	0 (0)	0 (0)	20 (95.2)	
**Grand Total +/R (%)**	**9 (21.4)**	42 (100)	42 (100)	**42 (100)**	**42 (100)**	**39 (92.9)**	**33 (78.6)**	**32 (76.2)**	**27 (64.3)**	**25 (59.5)**	**15 (35.7)**	**0 (0)**	**0 (0)**	**0 (0)**	**0 (0)**	**40/42 (95.2)**	

The most prevalent Spa types are presented in boldface font

*FOX* cefoxitin, *PEN* penicillin, *TET* tetracycline, *SXT*, *ERY* erythromycin, *CHL* chloramphenicol, *GEN* gentamycin, *CIP* ciprofloxacin, *CLI* clindamycin, *RIF* rifampicin, *VAN* vancomycin, *LZD* linezolid, *MDR* multidrug resistant –resistance to three or more classes of antimicrobials; *+* Positive, − Negative

### Spa types and SCC*mec* elements

**Fig. 1:**
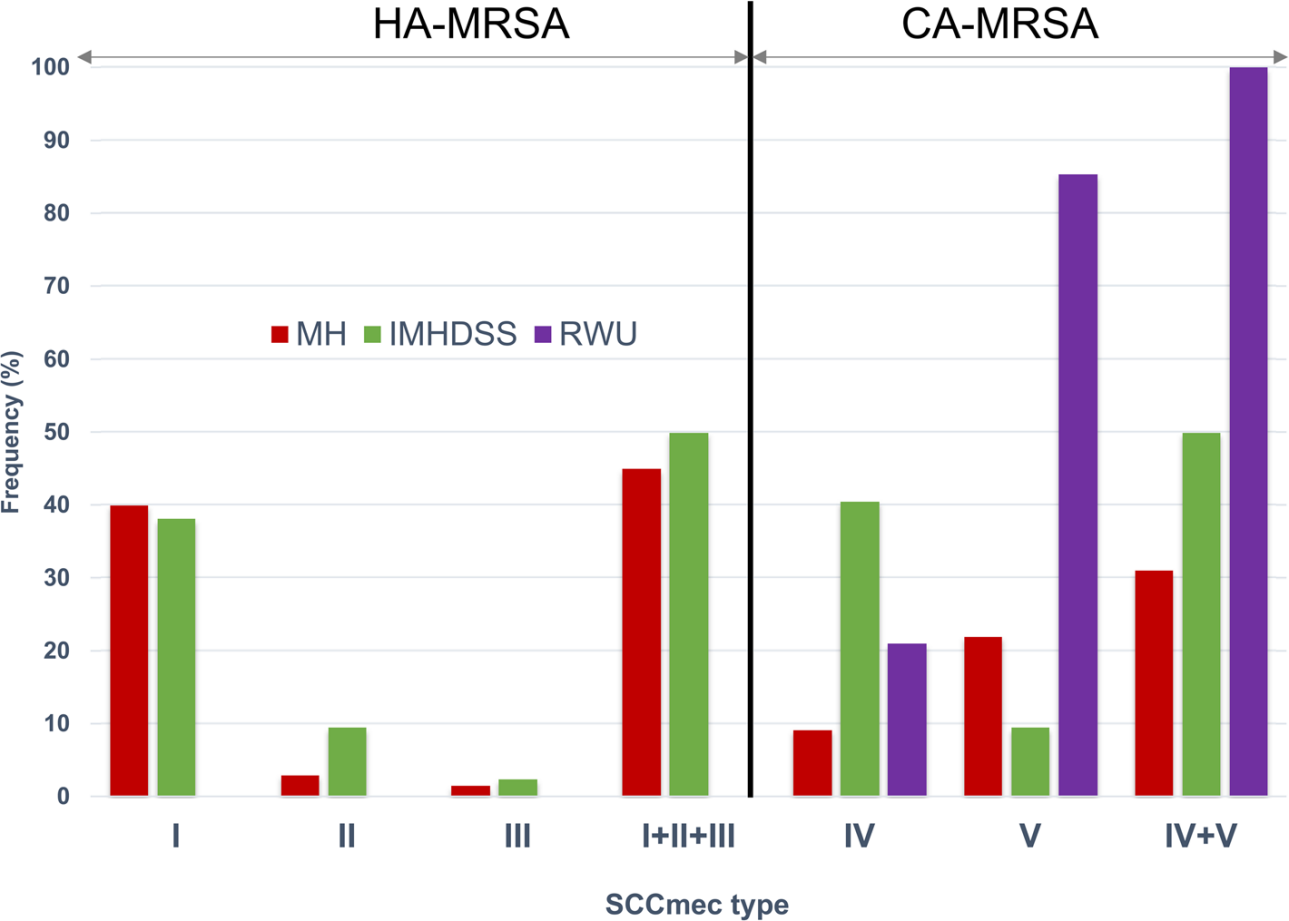
SCCmec types for MRSA isolates depicting coexistence between CA-MRSA and HA-MRSA in children under 5 years in rural eastern Uganda. Note that the categorization of MRSA isolates as CA-MRSA or HA-MRSA is based on their genetic background (i.e. SCC*mec* types) and not clinical/epidemiological associations. MH denotes Mulago National Referral Hospital; IMHDSS, Health & Demographic Surveillance Site; RWU, Rural Western Uganda

The most predominant SCC*mec* elements were SCC*mec* type IV (40.5%, 17/42) and SCC*mec* type I (38.1%, 16/42). SCC*mec* types II and V accounted for 4 isolates each (9.5%, 4/42) while SCC*mec* type III accounted for one isolate. The overall frequency of SCC*mec* types IV and V combined, which define the genetic background of MRSA isolates associated with the community, was 50% (21/42) implying the prevalence of CA-MRSA in children was 50% (21/42). This is relatively low compared to reported rates of CA-MRSA in the community. Furthermore, the overall frequency of SCC*mec* types I, II and III combined, which define the genetic trait of MRSA isolates associated with healthcare environments, was 50% (21/42) hence similar to SCC*mec* types IV and IV combined. The *PVL* gene prevalence was low (i.e. 21.4%, 9/42) and distributed equally in CA-MRSA and HA-MRSA, Table 1. Taken together, these data show that CA-MRSA and HA-MRSA coexist in children in the community in eastern Uganda, Table 1 & Fig. 1.

The observed coexistence between CA-MRSA and HA-MRSA in Uganda was first reported at Mulago Hospital in Kampala [22, 23] but not explored further. Therefore, we compared previously reported SCC*mec* types for isolates at Mulago Hospital [22, 23] and pastoral communities in western Uganda [30, 31] with SCC*mec* types for isolates from IMHDSS. Due to frequent interactions between healthcare personnel from the Mulago Hospital setting and community members in IMHDSS, we hypothesized that the SCC*mec* types’ distribution in the two settings would be similar. Indeed, there was no statistical significance (*P* = 0.1014) in the distribution of SCC*mec* types between the IMHSS and Mulago Hospital (Fig. 1) hence, CA-MRSA and HA-MRSA coexist in IMHDSS (community) and Mulago Hospital. Conversely, MRSA from pastoral communities in rural western Uganda carried only SCC*mec* types IV and V, Fig. 1.

A total of five genotypes (t002, t037, t064, t4353 and t12939) were detected among MRSA and the most predominant were t064 (19%, 8/42) and t037 (12%, 5/42). Thus, t064 and t037 are the prevalent spa types among CA-MRSA and HA-MRSA isolates respectively, Table 1 & Additional file 1: Figure S1. When we compared *spa* types with MRSA isolates from Mulago Hospital [22, 23] and pastoral communities in rural western Uganda [30, 31], we found that with the exception of t002 and t12939, spa types for MRSA isolates from IMHDSS were previously reported, Additional file 1: Figure S1. There were subtle differences in distribution of *spa* types with respect to setting e.g. *spa* types restricted to pastoral communities in rural western Uganda (circled in Additional file 1: Figure S1).

## Discussion

In this study, we found the MRSA carriage rate (5.7%) in children in eastern Uganda to be high and comparable to reported estimates for MRSA prevalence in adult populations in Uganda [7, 9, 23] and generally East Africa [33, 34]. Furthermore, we have shown that CA-MRSA and HA-MRSA strains coexist in the community in eastern Uganda and at Mulago hospital in Kampala city. Coexistence between CA-MRSA and HA-MRSA has been reported extensively in developed countries [16, 35–38] and beyond [35, 39–42] but few studies in Africa have explored it. Note, the terms ‘CA-MRSA’ and ‘HA-MRSA’ have also been used to describe the epidemiological and clinical features of MRSA e.g. the CDC case definition for CA-MRSA infection “Any MRSA infection diagnosed for an outpatient or within 48 h of hospitalization if the patient lacks the following health care-associated MRSA risk factors: hemodialysis, surgery, residence in a long-term care facility or hospitalization during the previous year, the presence of an indwelling catheter or a percutaneous device at the time of culture” [1], but such epidemiological/clinical definitions are also misleading [1, 14, 21]. While there is no consensus on interpretation of ‘HA-MRSA vs. CA-MRSA’, MRSA strains are genotypically distinguishable through a simple PCR assay, SCCmec typing. Currently there are nine SCC*mec* types –I, II, III, IVa, IVb, V, VI, VII, VIII and V_T_ [1]; types I, II and III are large and occur in HA-MRSA strains while types IV and V are smaller and occur in CA-MRSA strains [1, 2, 21].

There are several factors that could be fuelling coexistence between CA-MRSA and HA-MRSA in the community in eastern Uganda. First, previous exposure to antibiotics and/or health care facilities as the children were reported to be previously sick. The mothers reported that about one third of the children had been given ampicillin and co-trimoxazole and this could explain the high prevalence of MRSA and SXT resistance detected. It is important to note that over-treatment of children with antibiotics is common in Uganda as children suffer from 0.3 episodes of pneumonia every year [13]. Also, Uganda adopted the World Health Organization’s (WHO) integrated case management of childhood illnesses under which community health workers provide prompt treatment of malaria and bacterial infections for children less than 5 years of age. The WHO’s integrated case management guidelines are based on simple clinical signs to help health workers identify and manage malaria, pneumonia and other childhood illnesses in the community. Although they have been found to increase rational prescription of medicines, the WHO guidelines do not distinguish between viral, parasitic (excluding plasmodium) and bacterial infections implying that a significant number of children in the community receive antibiotics [13]. Thus, some MRSA strains in IMHDSS were indeed HA-MRSA perhaps arising from circulation of “escaped”/“feral” HA-MRSA strains in the community as a consequence of management of childhood infections at home [1].

The second factor underlying coexistence of CA-MRSA and HA-MRSA in children in eastern Uganda and generally at Mulago Hospital could be the interaction between healthcare workers and community members. Mathematical models of MRSA transmission have predicted that Hospital-Community interactions foster coexistence between CA-MRSA and HA-MRSA strains as the high discharge and hospitalization rates continuously cycle individuals between hospitals and the community [21]. Besides, other investigators have implicated frequent interaction between healthcare workers and community members in increasing the risk of colonization with MRSA in the community [17]. In Uganda, medical students, faculty, health care workers and researchers from the Mulago Hospital setting which includes Makerere University medical school use the IMHDSS as a site for disease surveillance, research and community-based medical education. The IMHDSS community members could also be colonized by HA-MRSA from nearby healthcare facilities as the IMHDSS is served by a government hospital, nine public health centers, three non-governmental organization hospitals, 122 drug shops and private clinics [24]. Furthermore, studies by Asiimwe et al. [30, 31] on MRSA carriage in pastoral communities in rural western Uganda provided additional support for coexistence between CA-MRSA and HA-MRSA in the IMHDSS and Mulago Hospital. MRSA isolates from pastoral communities in rural western Uganda carried only SCC*mec* types IV and V that are typical of CA-MRSA and SCC*mec* types I, II and III were not detected [30, 31] hence, CA-MRSA and HA-MRSA do not coexist in pastoral communities in rural western Uganda. In context of health service delivery in Uganda, this is understandable as pastoral communities are remote and often characterized with inadequate health service delivery.

Almost all MRSA isolates in this study were MDR. As HA-MRSA strains are associated with multiple resistance to non-β-lactam antimicrobial agents, perhaps their prevalence contributed to the observed high resistance to non-β-lactam antimicrobials. On the other hand, CA-MRSA strains are usually susceptible to non-β-lactam antimicrobials [1, 2] but in this study they were not. One explanation for the MDR phenotype and high resistance to non-β-lactam antimicrobials among CA-MRSA could be acquisition of drug resistance genes [27]. A similar trend of CA-MRSA being MDR has been observed elsewhere especially in Europe, Asia and the Americas [43–46]. Case in point is a study from a large veterinary teaching hospital in Costa Rica where nearly all CA-MRSA isolates investigated were MDR and carried SCC*mec* type IV [44].

Lastly, this study had a few limitations. First, the small number of MRSA isolates investigated implies that differences observed could be due to low frequencies of genotypes recorded. However, we sampled a larger population for recovery of MRSA compared to previous studies in Uganda [7, 9, 22, 23]. Second, for reasons already explained, we used isolate genotypic characteristics to classify MRSA isolates as our interest was in unambiguously identifying CA-MRSA and HA-MRSA. While important, clinical/epidemiological features were not considered as both CA-MRSA and HA-MRSA may occur in either setting i.e. community or hospital [14, 21].

## Conclusions

The MRSA carriage rate in children in rural eastern Uganda is high (5.7%) and comparable to estimates for a large urban teaching facility, Mulago National Referral Hospital, located in Uganda’s capital, Kampala. Importantly, HA-MRSA (mainly of *spa* type t037) and CA-MRSA (mainly of *spa* type t064) coexist in community and hospital settings in Uganda with no statistical significance for observed differences in rates. Because interaction between healthcare workers and community members contributes to presence of HA-MRSA in the community, standard hygiene measures should be reinforced to prevent cross-transmission at the IMHDSS. As well, due to the high proportion of MDR-MRSA detected, outpatient treatment of MRSA infections in eastern Uganda might be difficult.

## Additional file

Additional file 1: **Figure S1.** *Spa* types for MRSA isolates from children under 5 years in rural eastern Uganda. MH denotes Mulago National Referral Hospital; IMHDSS, Health & Demographic Surveillance Site; RWU, Rural Western Uganda. The circle signifies *spa* types that appear restricted to rural western Uganda. (TIFF 3173 kb)

## Data Availability

All data generated or analyzed during this study are included in this published article [and its supplementary information files].

## Abbreviations

CTAB: Cetrimonium bromide
IMHDSS: Iganga-Mayuge Health & Demographic Surveillance Site
*mecA*: Gene encoding the penicillin binding protein 2a (PBP2a)
MIC: Minimum inhibitory concentration
MRSA: Methicillin resistant *Staphylococcus aureus*
MSSA: Methicillin Susceptible *Staphylococcus aureus*
PCR: Polymerase chain reaction
PVL: Panton Valentine leukocidin
SCC*mec*: Staphylococcal cassette chromosome mec
Spa: Staphylococcal protein A
SXT: Trimethoprim/sulfamethoxazole

## References

[1] David MZ, Daum RS (2010). Community-associated methicillin-resistant Staphylococcus aureus: epidemiology and clinical consequences of an emerging epidemic. Clin Microbiol Rev.

[2] Kong Eric F., Johnson Jennifer K., Jabra-Rizk Mary Ann (2016). Community-Associated Methicillin-Resistant Staphylococcus aureus: An Enemy amidst Us. PLOS Pathogens.

[3] Herrmann M, Abdullah S, Alabi A, Alonso P, Friedrich AW, Fuhr G, Germann A, Kern WV, Kremsner PG, Mandomando I (2013). Staphylococcal disease in Africa: another neglected 'tropical' disease. Future Microbiol.

[4] Schaumburg F, Alabi AS, Peters G, Becker K (2014). New epidemiology of Staphylococcus aureus infection in Africa. Clin Microbiol Infect.

[5] Jacob ST, Moore CC, Banura P, Pinkerton R, Meya D, Opendi P, Reynolds SJ, Kenya-Mugisha N, Mayanja-Kizza H, Scheld WM (2009). Severe Sepsis in two Ugandan hospitals: a prospective observational study of management and outcomes in a predominantly HIV-1 infected population. PLoS One.

[6] Mugalu J, Nakakeeto MK, Kiguli S, Kaddu-Mulindwa DH (2006). Aetiology, risk factors and immediate outcome of bacteriologically confirmed neonatal septicaemia in Mulago hospital, Uganda. Afr Health Sci.

[7] Ojulong J, Mwambu T, Joloba M, Bwanga F, Kaddu-Mulindwa D (2009). Relative prevalence of methicilline resistant Staphylococcus aureus and its susceptibility pattern in mulago hospital, Kampala, Uganda. Tanzan J Health Res.

[8] Seni J, Najjuka CF, Kateete DP, Makobore P, Joloba ML, Kajumbula H, Kapesa A, Bwanga F (2013). Antimicrobial resistance in hospitalized surgical patients: a silently emerging public health concern in Uganda. BMC Res Notes.

[9] Anguzu JR, Olila D (2007). Drug sensitivity patterns of bacterial isolates from septic post-operative wounds in a regional referral hospital in Uganda. Afr Health Sci.

[10] Kizito M, Mworozi E, Ndugwa C, Serjeant GR (2007). Bacteraemia in homozygous sickle cell disease in Africa: is pneumococcal prophylaxis justified?. Arch Dis Child.

[11] Bachou H, Tylleskar T, Kaddu-Mulindwa DH, Tumwine JK (2006). Bacteraemia among severely malnourished children infected and uninfected with the human immunodeficiency virus-1 in Kampala, Uganda. BMC Infect Dis.

[12] Falagas ME, Karageorgopoulos DE, Leptidis J, Korbila IP (2013). MRSA in Africa: filling the global map of antimicrobial resistance. PLoS One.

[13] Rutebemberwa E, Mpeka B, Pariyo G, Peterson S, Mworozi E, Bwanga F, Källander K (2015). High prevalence of antibiotic resistance in nasopharyngeal bacterial isolates from healthy children in rural Uganda: a cross-sectional study. Ups J Med Sci.

[14] Choo EJ (2017). Community-associated methicillin-resistant Staphylococcus aureus in nosocomial infections. Infect Chemother.

[15] Abdulgader SM, Shittu AO, Nicol MP, Kaba M (2015). Molecular epidemiology of methicillin-resistant Staphylococcus aureus in Africa: a systematic review. Front Microbiol.

[16] Hudson LO, Murphy CR, Spratt BG, Enright MC, Terpstra L, Gombosev A, Hannah P, Mikhail L, Alexander R, Moore DF (2012). Differences in methicillin-resistant Staphylococcus aureus strains isolated from pediatric and adult patients from hospitals in a large county in California. J Clin Microbiol.

[17] Prosperi M, Veras N, Azarian T, Rathore M, Nolan D, Rand K, Cook RL, Johnson J, Morris JG, Salemi M (2013). Molecular epidemiology of community-associated methicillin-resistant Staphylococcus aureus in the genomic era: a cross-sectional study. Sci Rep.

[18] Monecke S, Coombs G, Shore AC, Coleman DC, Akpaka P, Borg M, Chow H, Ip M, Jatzwauk L, Jonas D (2011). A field guide to pandemic, epidemic and sporadic clones of methicillin-resistant Staphylococcus aureus. PLoS One.

[19] David MZ, Cadilla A, Boyle-Vavra S, Daum RS (2014). Replacement of HA-MRSA by CA-MRSA infections at an academic medical center in the midwestern United States, 2004-5 to 2008. PLoS One.

[20] Nimmo GR, Coombs GW (2008). Community-associated methicillin-resistant Staphylococcus aureus (MRSA) in Australia. Int J Antimicrob Agents.

[21] Kouyos R, Klein E, Grenfell B (2013). Hospital-community interactions Foster coexistence between methicillin-resistant strains of Staphylococcus aureus. PLoS Pathog.

[22] Kateete DP, Namazzi S, Okee M, Okeng A, Baluku H, Musisi NL, Katabazi FA, Joloba ML, Ssentongo R, Najjuka FC (2011). High prevalence of methicillin resistant Staphylococcus aureus in the surgical units of Mulago hospital in Kampala, Uganda. BMC Res Notes.

[23] Seni J, Bwanga F, Najjuka CF, Makobore P, Okee M, Mshana SE, Kidenya BR, Joloba ML, Kateete DP (2013). Molecular characterization of Staphylococcus aureus from patients with surgical site infections at Mulago Hospital in Kampala, Uganda. PloS one.

[24] Kalyango JN, Alfven T, Peterson S, Mugenyi K, Karamagi C, Rutebemberwa E (2013). Integrated community case management of malaria and pneumonia increases prompt and appropriate treatment for pneumonia symptoms in children under five years in eastern Uganda. Malar J.

[25] Kateete DP, Kimani CN, Katabazi FA, Okeng A, Okee MS, Nanteza A, Joloba ML, Najjuka FC (2010). Identification of Staphylococcus aureus: DNase and mannitol salt agar improve the efficiency of the tube coagulase test. Ann Clin Microbiol Antimicrob.

[26] CLSI C: Performance standards for antimicrobial susceptibility testing. *Clinical and Laboratory Standards Institute (M100eS22)* 2012 (s22nd Informational Supplement).

[27] Nimmo GR, Bergh H, Nakos J, Whiley D, Marquess J, Huygens F, Paterson DL (2013). Replacement of healthcare-associated MRSA by community-associated MRSA in Queensland: confirmation by genotyping. J Infect.

[28] Coombs GW, Daley DA, Lee YT, Pang S, Bell JM, Turnidge JD (2018). Australian group on antimicrobial resistance (AGAR) Australian Staphylococcus aureus Sepsis outcome Programme (ASSOP) annual report 2015. Commun Dis Intell.

[29] Boye K, Bartels MD, Andersen IS, Moller JA, Westh H (2007). A new multiplex PCR for easy screening of methicillin-resistant Staphylococcus aureus SCCmec types I-V. Clin Microbiol Infect.

[30] Asiimwe BB, Baldan R, Trovato A, Cirillo DM (2017). Prevalence and molecular characteristics of Staphylococcus aureus, including methicillin resistant strains, isolated from bulk can milk and raw milk products in pastoral communities of south-West Uganda. BMC Infect Dis.

[31] Asiimwe BB, Baldan R, Trovato A, Cirillo DM (2017). Molecular epidemiology of Panton-valentine Leukocidin-positive community-acquired methicillin resistant Staphylococcus aureus isolates in pastoral communities of rural south western Uganda. BMC Infect Dis.

[32] Harmsen D, Claus H, Witte W, Rothganger J, Turnwald D, Vogel U (2003). Typing of methicillin-resistant Staphylococcus aureus in a university hospital setting by using novel software for spa repeat determination and database management. J Clin Microbiol.

[33] Joachim A, Moyo SJ, Nkinda L, Majigo M, Mmbaga E, Mbembati N, Aboud S, Lyamuya EF (2017). Prevalence of methicillin-resistant Staphylococcus aureus carriage on admission among patients attending regional hospitals in Dar Es Salaam, Tanzania. BMC Res Notes.

[34] Aiken AM, Mutuku IM, Sabat AJ, Akkerboom V, Mwangi J, Scott JAG, Morpeth SC, Friedrich AW, Grundmann H (2014). Carriage of Staphylococcus aureus in Thika level 5 hospital, Kenya: a cross-sectional study. Antimicrob Resist Infect Control.

[35] Hu Q, Cheng H, Yuan W, Zeng F, Shang W, Tang D, Xue W, Fu J, Zhou R, Zhu J (2015). Panton-valentine leukocidin (PVL)-positive health care-associated methicillin-resistant Staphylococcus aureus isolates are associated with skin and soft tissue infections and colonized mainly by infective PVL-encoding bacteriophages. J Clin Microbiol.

[36] Lee TM, Yang MC, Yang TF, Lee PL, Chien HI, Hsueh JC, Chang SH, Hsu CH, Chien ST (2015). Molecular Characterization of Community- and Healthcare-Associated Methicillin-Resistant *Staphylococcus aureus* Isolates in Southern Taiwan. Microb Drug Resist (Larchmont, NY).

[37] Miura Y, Yamaguchi T, Nakamura I, Koyama S, Tamai K, Okanda T, Matsumoto T (2018). Epidemiological trends observed from molecular characterization of methicillin-resistant Staphylococcus aureus isolates from blood cultures at a Japanese University hospital**,** 2012-2015. Microbial Drug Resis (Larchmont, NY).

[38] Tenover FC, Tickler IA, Goering RV, Kreiswirth BN, Mediavilla JR, Persing DH (2012). Characterization of nasal and blood culture isolates of methicillin-resistant Staphylococcus aureus from patients in United States hospitals. Antimicrob Agents Chemother.

[39] Egyir B, Guardabassi L, Sørum M, Nielsen SS, Kolekang A, Frimpong E, Addo KK, Newman MJ, Larsen AR (2014). Molecular epidemiology and antimicrobial susceptibility of clinical Staphylococcus aureus from healthcare institutions in Ghana. PLoS One.

[40] Moodley A, Oosthuysen WF, Dusé AG, Marais E (2010). Molecular characterization of clinical methicillin-resistant <em>Staphylococcus aureus</em> isolates in South Africa. J Clin Microbiol.

[41] Shittu A, Oyedara O, Abegunrin F, Okon K, Raji A, Taiwo S, Ogunsola F, Onyedibe K, Elisha G (2012). Characterization of methicillin-susceptible and -resistant staphylococci in the clinical setting: a multicentre study in Nigeria. BMC Infect Dis.

[42] Shittu AO, Okon K, Adesida S, Oyedara O, Witte W, Strommenger B, Layer F, Nübel U (2011). Antibiotic resistance and molecular epidemiology of Staphylococcus aureus in Nigeria. BMC Microbiol.

[43] Earls MR, Kinnevey PM, Brennan GI, Lazaris A, Skally M, O’Connell B, Humphreys H, Shore AC, Coleman DC (2017). The recent emergence in hospitals of multidrug-resistant community-associated sequence type 1 and spa type t127 methicillin-resistant Staphylococcus aureus investigated by whole-genome sequencing: implications for screening. PLoS One.

[44] Rojas I, Barquero-Calvo E, van Balen JC, Rojas N, Muñoz-Vargas L, Hoet AE (2017). High Prevalence of Multidrug-Resistant Community-Acquired Methicillin-Resistant *Staphylococcus aureus* at the Largest Veterinary Teaching Hospital in Costa Rica. Vector Borne Zoonotic Dis (Larchmont, NY).

[45] Wang L, Liu Y, Yang Y, Huang G, Wang C, Deng L, Zheng Y, Fu Z, Li C, Shang Y (2012). Multidrug-resistant clones of community-associated meticillin-resistant Staphylococcus aureus isolated from Chinese children and the resistance genes to clindamycin and mupirocin. J Med Microbiol.

[46] Wilson J, Lawson KA, Frei CR, Lee GC, Dallas SD, Wang Y, Olsen RJ (2017). Emerging multidrug resistance in community-associated Staphylococcus aureus involved in skin and soft tissue infections and nasal colonization. J Antimicrob Chemother.

