# Supplementary figures and images for: CA-MRSA and HA-MRSA coexist in community and hospital settings in Uganda
Source: Antimicrob Resist Infect Control. 2019 Jun 3;8:94. doi: 10.1186/s13756-019-0551-1 (PMC6547506; doi:10.1186/s13756-019-0551-1)

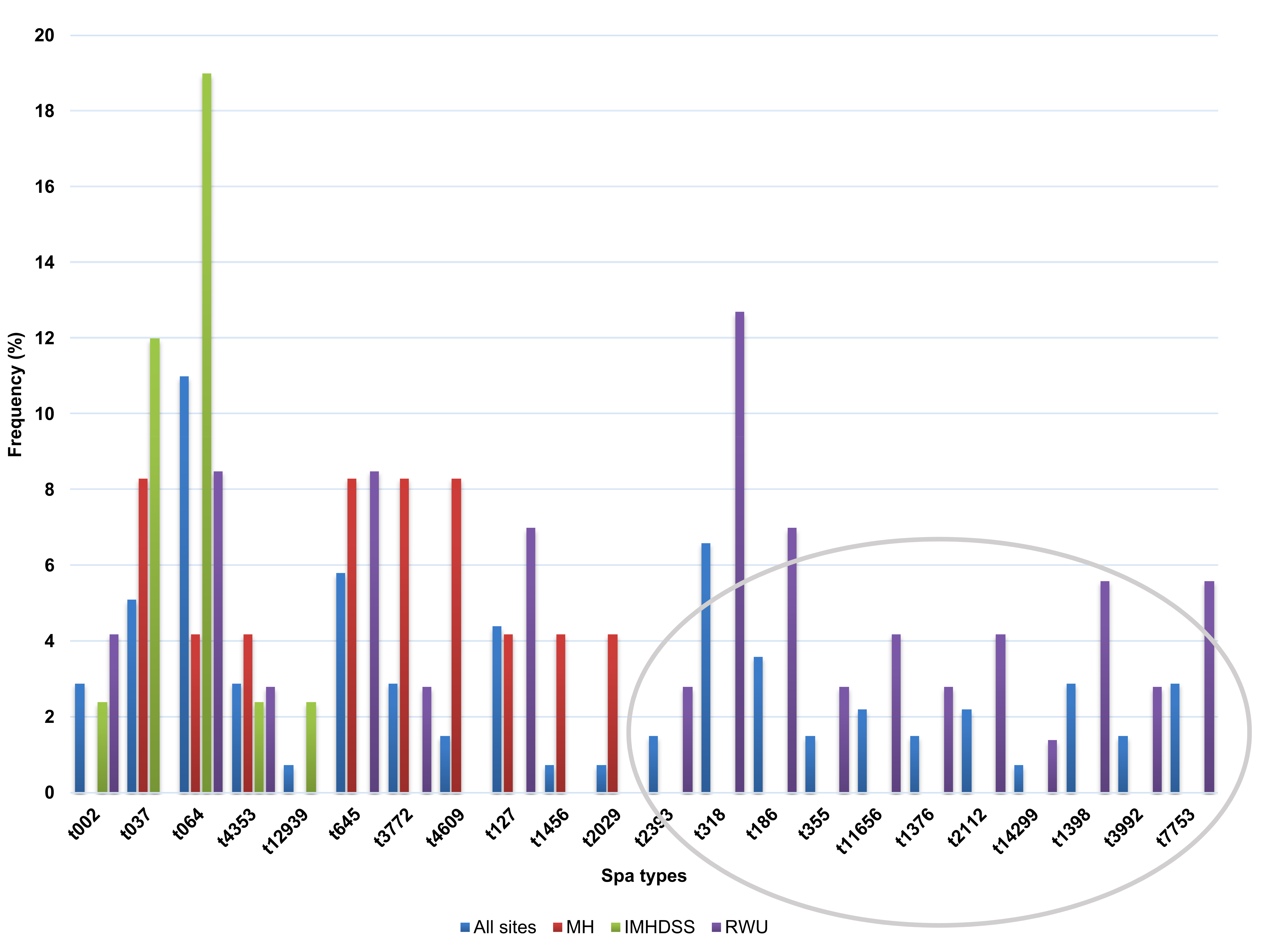

Supplement: Supplementary file 1 — Figure S1. Spa types for MRSA isolates from children under 5 years in rural eastern Uganda. MH denotes Mulago National Referral Hospital; IMHDSS, Health & Demographic Surveillance Site; RWU, Rural Western Uganda. The circle signifies spa types that appear restricted to rural western Uganda. (TIFF 3173 kb) [file 13756_2019_551_MOESM1_ESM.tiff]
